# Comparative Effects of Phosphoenolpyruvate, a Glycolytic Intermediate, as an Organ Preservation Agent with Glucose and N-Acetylcysteine against Organ Damage during Cold Storage of Mouse Liver and Kidney

**DOI:** 10.1155/2013/375825

**Published:** 2013-12-05

**Authors:** Yoichi Ishitsuka, Yusuke Fukumoto, Yuki Kondo, Mitsuru Irikura, Daisuke Kadowaki, Yuki Narita, Sumio Hirata, Hiroshi Moriuchi, Toru Maruyama, Naotaka Hamasaki, Tetsumi Irie

**Affiliations:** ^1^Department of Clinical Chemistry and Informatics, Graduate School of Pharmaceutical Sciences, Kumamoto University, 5-1 Oe-honmachi, Chuo-ku, Kumamoto 862-0973, Japan; ^2^Department of Biopharmaceutics, Graduate School of Pharmaceutical Sciences, Kumamoto University, 5-1 Oe-honmachi, Chuo-ku, Kumamoto 862-0973, Japan; ^3^Center for Clinical Pharmaceutical Sciences, Faculty of Pharmaceutical Sciences, Kumamoto University, 5-1 Oe-honmachi, Chuo-ku, Kumamoto 862-0973, Japan; ^4^Department of Clinical Pharmacy, Faculty of Pharmaceutical Science, Sojo University, 4-22-1 Ikeda, Nishi-ku, Kumamoto 860-0082, Japan; ^5^Department of Clinical Chemistry and Laboratory Medicine, Faculty of Pharmaceutical Sciences, Nagasaki International University, Sasebo 859-3298, Japan

## Abstract

We evaluated the usefulness of phosphoenolpyruvate (PEP), a glycolytic intermediate with antioxidative and energy supplementation potentials, as an organ preservation agent. Using *ex vivo* mouse liver and kidney of a static cold storage model, we compared the effects of PEP against organ damage and oxidative stress during cold preservation with those of glucose or N-acetylcysteine (NAC). Lactate dehydrogenase (LDH) leakage, histological changes, and oxidative stress parameters (measured as thiobarbituric acid reactive substance and glutathione content) were determined. PEP (100 mM) significantly prevented an increase in LDH leakage, histological changes, such as tubulonecrosis and vacuolization, and changes in oxidative stress parameters during 72 h of cold preservation in mouse liver. Although glucose (100 mM) partly prevented LDH leakage and histological changes, no effects against oxidative stress were observed. By contrast, NAC inhibited oxidative stress in the liver and did not prevent LDH leakage or histological changes. PEP also significantly prevented kidney damage during cold preservation in a dose-dependent manner, and the protective effects were superior to those of glucose and NAC. We suggest that PEP, a functional carbohydrate with organ protective and antioxidative activities, may be useful as an organ preservation agent in clinical transplantation.

## 1. Introduction

Clinical transplantation, such as liver or kidney transplantation, is the only life-saving therapy for some severe diseases, such as end-stage kidney, liver failure, or chronic end-stage liver diseases. It is important to conserve the condition of the graft before transplantation because damage of the graft during cold preservation is one of the major risk factors for graft dysfunction. To solve this, organ preservation solutions, such as Euro-Collins solution and University of Wisconsin solution, are used [1, 2]. These solutions are generally composed of electrolytes and buffers, with carbohydrates, such as glucose and trehalose, added to prevent energy loss and increase the osmotic pressure of the solution. In addition, to inhibit the generation of reactive oxygen species during cold preservation and ischemia/reperfusion, antioxidants, glutathione and N-acetylcysteine, are added to the University of Wisconsin solution and ET-Kyoto solution, respectively [3, 4]. Although these solutions are essential for clinical transplantation, they have several disadvantages, including chemical instability and high viscosity. Additionally, they are not costeffective for short-term storage [5]. Therefore, development of a new and effective reagent for organ preservation is critical.

Phosphoenolpyruvate (PEP), an intermediate of glycolysis and gluconeogenesis, may have attractive biological functions. PEP can penetrate the cell membrane and transfer its high-energy phosphate group to ADP to replenish intracellular ATP levels [6]. It has been reported that PEP improves the energy status of the heart [7] and skeletal muscle [8] after ischemia and that of the liver after ischemia-reperfusion [9]. Recently, we demonstrated that PEP has scavenging potentials against reactive oxygen species, such as hydroxyl radicals and hydrogen peroxide, which contribute to pathophysiological damage as inducers of oxidative stress in a diseased state [10, 11]. We also elucidated that PEP drastically attenuates cellular injury induced by hydrogen peroxide and 2-deoxy-D-glucose, a glycolysis inhibitor, in the porcine proximal kidney tubular cell line LLC-PK1, human hepatocyte cell line HepG2 cells, and HeLa cells [10–12]. In addition, we demonstrated that PEP attenuates the elevation of aminotransferase leakage during organ preservation, histological changes, changes in oxidative stress parameters (measured as thiobarbituric acid reactive substance and glutathione content), and ATP content induced by 72 h of cold preservation of mouse liver in an *ex vivo* cold preservation system [11]. These *in vitro* and *ex vivo* results suggest that PEP is a functional carbohydrate metabolite with cytoprotective and antioxidative activities and may be suitable for organ cold preservation in a clinical transplantation setting. However, it was unclear whether the organ protective effects of PEP were superior to those of other organ preservation agents, such as glucose or NAC, in a mouse liver *ex vivo* system or whether PEP could exert organ protective effects in other organs, such as kidney, during cold preservation.

Based on these facts, this study was conducted to examine whether PEP can prevent renal damage during cold storage by quantifying cellular injury and histological changes in a mouse kidney *ex vivo* system. In addition, to evaluate the usefulness of PEP as an organ preservation agent, we compared the protective effects of PEP with those of components used in organ preservation solutions, such as glucose or NAC.

## 2. Materials and Methods

### 2.1. Materials

Sodium phosphoenolpyruvate monohydrate was kindly donated by Ube Kousan (Yamaguchi, Japan). A lactate dehydrogenase assay kit was purchased from ARKRAY, Inc. (Kyoto, Japan). Glucose and NAC were obtained from Nacalai Tesque, Inc., (Kyoto, Japan) and Sigma (St. Louis, MO, USA), respectively. A TBARS assay kit and BIOXYTECH GSH/GSSG-412 were purchased from Cayman Chemical Co. (Ann Arbor, MI, USA) and Percipio Biosciences, Inc. (Manhattan Beach, CA, USA), respectively. All other reagents and solvents were of reagent grade. Deionized and distilled biopure grade water was used throughout the study.

### 2.2. *Ex Vivo *Cold-Preservation Liver and Kidney Mouse Model

The *ex vivo* cold storage liver model was developed as previously described [11]. Phosphate-buffered saline (PBS, pH 7.4), 1 mM, 10 mM, and 100 mM PEP, 100 mM glucose, and 10 mM NAC dissolved in PBS were used as the preservation solutions. C57BL/6J mice (male, 25–30 g, purchased from CLEA Japan, Inc., Konan, Japan) were used in this study. Mice were anaesthetized with diethyl ether inhalation, and then a midline abdominal incision was made. The portal vein was cannulated using a 26 G needle. The systemic circulation was then perfused with each solution, and the inferior vena cava was incised. Subsequently, the liver and kidneys were carefully removed and preserved at 4°C for 72 h in each solution. All experimental procedures conformed to the animal experiment guidelines of the Committee for the Ethics on Animal Experiments of Kumamoto University (Approval number C24-269).

The organ damage was assessed by elevation of LDH in the preservation solution and histological analysis. The level of LDH was measured in the samples of the storage solution at 24, 48, and 72 h using a bioanalyzer (SPOTCHEM EZ SP-4430; ARKRAY, Inc.). After 72 h of preservation, the liver and kidney were used for histological analysis. The samples were fixed in 10% neutralized buffered formalin, embedded in paraffin, and sliced (3 mm thickness), and the slides were stained with hematoxylin and eosin. The samples were photographed and analyzed using a microscope system (BIOREVO BZ-9000; KEYENCE Co., Osaka, Japan). A liver and kidney that had not been subjected to cold storage served as a control for the histological analysis.

Oxidative stress parameters, including thiobarbituric acid reactive substance (TBARS) and reduced and oxidized glutathione (GSH and GSSG, resp.), in the liver homogenates were measured. After 72 h of cold preservation, the liver tissue (approximately 50 mg) and 500 mL of radioimmunoprecipitation assay (RIPA) buffer were mixed and homogenized under ice-cold conditions. The sample was centrifuged (1600 ×g, 10 min, 4°C), and the concentrations of TBARS and total proteins in the supernatant were measured using the TBARS assay kit and BCA Protein Assay Kit, respectively. For the measurements of GSH and GSSG, the liver tissues were weighed and mixed with 5% metaphosphoric acid solution at a 1 : 5 ratio (w/v) and homogenized under ice-cold conditions. The sample was centrifuged (1600 ×g, 10 min, 4°C), and the concentrations of GSH and GSSG were measured using a BIOXYTECH GSH assay kit.

### 2.3. Statistical Analysis

The results are expressed as the mean ± SEM. Statistical analysis was performed using GraphPad Prism ver. 5.01 (GraphPad Software, San Diego, CA, USA). Two-way analysis of variance was performed to examine the statistical significance of the data. When significant differences (*P* < 0.05) were identified, the data were further analyzed using Bonferroni's multiple comparison test.

## 3. Results

### 3.1. Effects of PEP, Glucose, or NAC on the Leakage of LDH in Preservation Media and Histological Changes in Mice Liver Induced by Cold Storage

**Figure 1:**
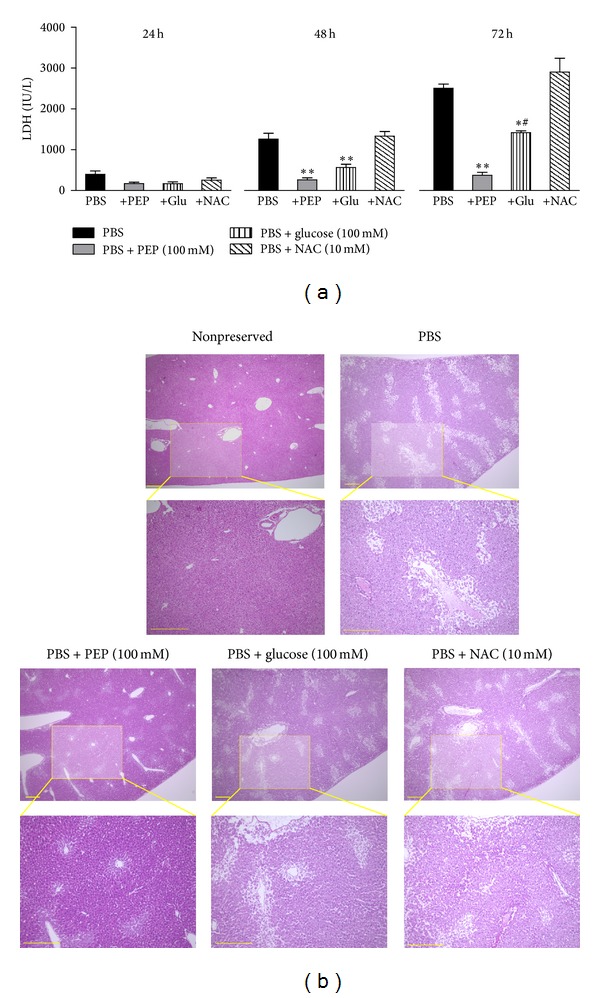
Effects of PEP, glucose, or NAC on mouse liver damage during cold preservation. (a) Changes in LDH concentrations in the preservation solutions; PBS (*n* = 3), PBS + 100 mM PEP (*n* = 3), PBS + 100 mM glucose (*n* = 3), and PBS + 10 mM NAC (*n* = 3). Each bar represents the mean ± SEM. *P < 0.05 and **P < 0.01, compared with the PBS group. ^#^ *P* < 0.05, compared with the PBS + 100 mM PEP group. (b) Histological pictures of the liver 72 h after cold preservation. The liver tissues were fixed with 10% buffered formalin, embedded in paraffin, sectioned, and stained with hematoxylin-eosin. Nonpreserved fresh liver served as a control. Scale bar = 300 *μ*m.

The time course of LDH levels in the preservation solutions is shown in Figure 1(a). The concentrations of LDH in the preserved solution were increased with storage time in the PBS groups. The increase in LDH levels was significantly prevented by treatment with 100 mM PEP, which was identified in our previous study as an adequate dose for mouse liver preservation [11]. Although the treatment with 100 mM glucose also inhibited increases in LDH levels, significant differences were observed between the glucose and PEP groups undergoing 72 h cold preservation. Treatment with 10 mM NAC did not prevent increases in LDH leakage during cold preservation.

The representative pictures of the liver histology after 72 h of preservation in each solution are shown in Figure 1(b). Significant colliquative necrosis of centrilobular hepatocytes, hypertrophy, vacuolization in middle and peripheral areas of the lobules, severe disappearance of hepatic cords, and deranged hepatic lobules that were poorly stained by eosin were observed in the PBS-preserved mouse liver. These histological changes were drastically attenuated by the treatment of 100 mM PEP. Although the treatment with 100 mM glucose also prevented these histological changes, the effect was inferior to that induced by PEP. Treatment with 10 mM NAC also did not show any effects on these histological changes.

### 3.2. Effect of PEP on the Changes in Oxidative Stress Parameters in Liver Homogenates

**Figure 2:**
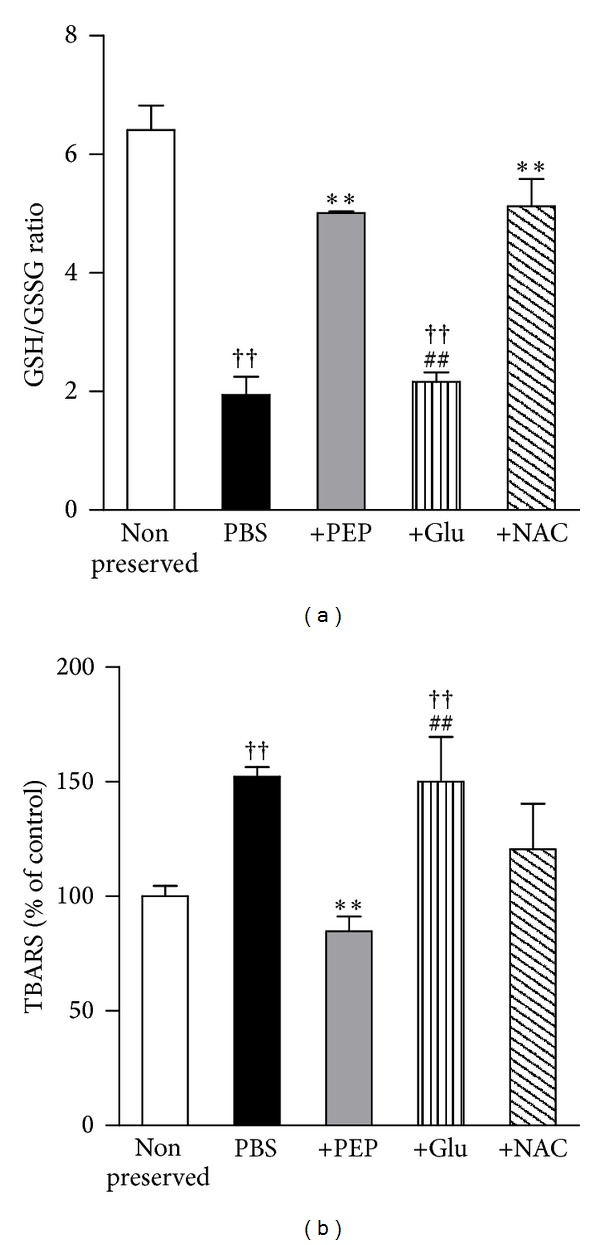
Changes in oxidative stress parameters, (a) GSH/GSSG ratio and (b) TBARS, in cold-preserved mouse liver. The study group was divided into five groups as follows: nonpreserved fresh liver, as a control (*n* = 3), PBS (*n* = 3), PBS + 100 mM PEP (*n* = 3), PBS + 100 mM glucose (*n* = 3), and PBS + 10 mM NAC (*n* = 3). Seventy-four hours after storage, the liver tissues were homogenized and analyzed. Each bar represents the mean ± SEM. **P < 0.01, compared with the PBS group; ^††^ *P* < 0.01, compared with the nonpreserved group; ^##^ *P* < 0.01, compared with the PBS + 100 mM PEP group.

The changes in oxidative stress parameters, including an increase in TBARS and a decrease in the GSH/GSSG ratio, are shown in Figure 2. The liver homogenate treated with PBS for 72 h showed approximately a 145% increase in TBARS levels and a 60% decrease in the GSH/GSSG ratio compared with those of the nonpreserved fresh liver. Significant decreases were observed in the oxidative stress parameters in the liver homogenates of the 100 mM PEP group compared with the PBS group. In contrast, treatment with 100 mM glucose did not show any effects against changes in the oxidative stress parameters compared with the PBS group. Treatment with 10 mM NAC tended to inhibit changes in the oxidative stress parameters; a significant difference in the GSH/GSSG ratio was observed between the NAC group and the PBS group.

### 3.3. Effect of PEP Treatment on the Kidney Damage during Cold Preservation

**Figure 3:**
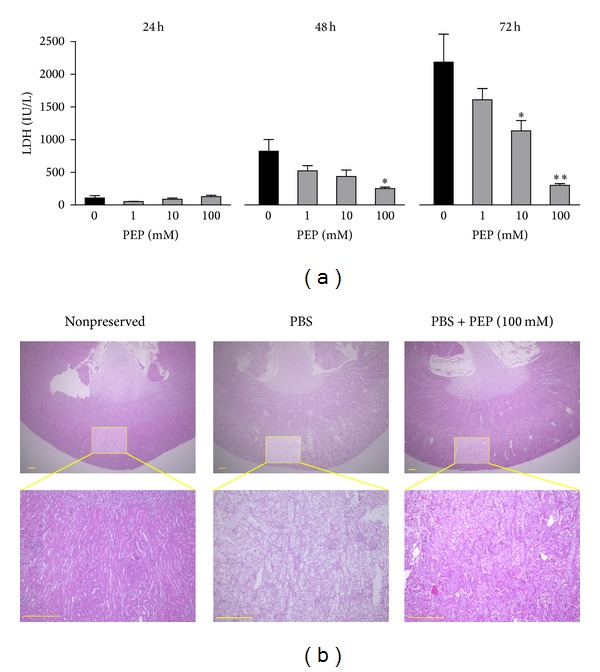
Effects of PEP on mouse kidney damage during cold preservation. (a) Changes in LDH concentrations in the preservation solutions; PBS (*n* = 3), PBS + 1 mM PEP (*n* = 4), + 10 mM PEP (*n* = 4), and + 100 mM PEP (*n* = 4). Each bar represents the mean ± SEM. *P < 0.05 and **P < 0.01, compared with the PBS group. (b) Histological pictures of the kidney 72 h after cold preservation. The liver tissues were fixed with 10% buffered formalin, embedded in paraffin, sectioned, and stained with hematoxylin-eosin. Nonpreserved fresh kidney served as a control. Scale bar = 300 *μ*m.

In addition to the hepatoprotective effects, we evaluated the renal protective effects of PEP during cold preservation. The time course of LDH levels in the preservation solutions is shown in Figure 3. When the kidney was preserved with 1–100 mM PEP, the increase in LDH was drastically inhibited in a dose-dependent manner (Figure 3(a)). Significant differences were observed between the PBS group and the 100 mM PEP group in 48 and 72 h cold preservation. In addition, a significant difference was observed in the 10 mM PEP group compared with the PBS group 72 h after cold storage. As shown in Figure 3(b), significant tubulonecrosis, vacuolization, and deranged renal tissue that was poorly stained by eosin were observed in the PBS-preserved mouse kidney. In contrast, these histological changes were reduced by the 100 mM PEP treatment.

**Figure 4:**
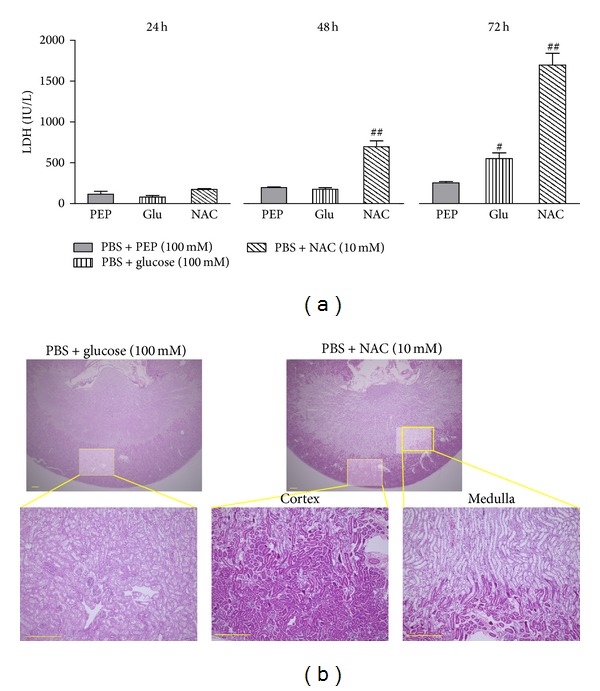
Comparative effects of PEP, glucose, and NAC on mouse kidney damage during cold preservation. (a) Changes in LDH concentrations in the preservation solutions; PBS + 100 mM PEP (*n* = 3), PBS + 100 mM glucose (*n* = 3), and PBS + 10 mM NAC (*n* = 4). Each bar represents the mean ± SEM. ^#^ *P* < 0.05, ^##^ *P* < 0.01, compared with the PBS + 100 mM PEP group. (b) Histological pictures of the kidney 72 h after cold preservation. The liver tissues were fixed with 10% buffered formalin, embedded in paraffin, sectioned, and stained with hematoxylin-eosin. Scale bar = 300 *μ*m.

As shown in Figure 4, we compared the effects of PEP with glucose or NAC against renal damage during cold preservation. In the 100 mM PEP group, no statistically significant changes in LDH concentrations as a function of time were observed during cold preservation. In contrast, a significant increase was observed after 72 h of preservation compared with that after 24 h of preservation in the glucose-treated group. Moreover, a significant increase in LDH concentrations after 48 h and 72 h of preservation in the NAC-containing solution was observed compared with that 24 h. Significant differences were observed between the PEP and glucose groups after 72 h of preservation and between the PEP group and the NAC group after 48 h and 72 h of preservation (Figure 4(a)). The histological pictures of the kidney after 72 h of preservation in each preservation solution are shown in Figure 4(b). The histological changes related to cold preservation were partially reduced by glucose treatment. In the NAC treatment group, tissue that was poorly stained by eosin and vacuolization were observed in the renal medulla. Meanwhile, noticeable changes were not observed in the renal cortex compared with the medulla.

## 4. Discussion

In this study, we demonstrated that PEP, an intermediate of glycolysis, markedly inhibited mouse liver and kidney injury during cold preservation using an *ex vivo* mouse model as measured by LDH release and histological examination, and the organ protective effects were superior to those of glucose and NAC, components of existing preservation solutions for clinical organ transplantation. These results indicate that PEP can reduce organ damage related to cold storage and suggest that PEP is a superior organ preservation agent compared with existing components.

In the previous study, we demonstrated that PEP can prevent organ damage and oxidative stress changes induced by cold preservation in an *ex vivo *mouse liver model and an *in vitro* HepG2 cell model [11]. In addition, we showed that PEP showed radical scavenging effects against reactive oxygen and nitrogens, such as hydroxyl radicals, hydrogen peroxide, and peroxynitrite [10, 11]. Based on these facts, we considered that the protective effects of PEP against organ damage (measured by LDH concentration) induced by cold preservation may be exerted through antioxidative mechanisms. In this study, PEP also prevented both organ damage and oxidative stress changes induced by cold preservation in mice liver. However, NAC, a radical scavenger, did not inhibit organ damage when it could prevent oxidative stress changes induced by cold preservation. Moreover, glucose attenuated organ damage without the inhibition of oxidative stress. These results indicate that oxidative stress did not directly contribute to organ damage during cold preservation in this *ex vivo* model. In general, the carbohydrate contained in preservation solutions used for clinical transplantation, such as glucose or trehalose, may maintain the energy state and osmotic pressure and protect the organ against cold preservation [5]. Therefore, the organ protective effects of PEP may be exerted through the same mechanisms as those of carbohydrates.

Glucose has been used for clinical transplantation as a component of the Euro-Collins solution, a representative preservation solution. The results of this study indicate superior effects of PEP compared with glucose against organ damage induced by cold preservation and suggest the usefulness of PEP as an organ preservation agent in clinical transplantation. In addition to the general effects as a carbohydrate, the antioxidative effects of PEP will be advantageous when used as an organ preservation agent in clinical transplantation. In transplantation, cold preservation and warm reperfusion of the graft can induce oxidative stress and lead to dysfunction and acute rejection of the graft [13]. The University of Wisconsin solution contains glutathione and allopurinol, whereas the ET-Kyoto solution contains NAC. These preservation solutions seem to achieve better results than other preservation solutions [14–16]. PEP may be an attractive substance with the potential to act as both a carbohydrate and an antioxidant.

In this study, we demonstrated that PEP could protect the kidney from damage induced during cold preservation, which is also the case for the liver. These results confirm organ protection by PEP and support its usefulness as an organ preservation solution. To establish the efficacy of PEP as a component of organ preservation solutions, it is important to clarify whether PEP-containing organ preservation solutions are safe for both the allograft and the recipient. Therefore, further basic and clinical studies are warranted.

In summary, PEP drastically attenuated organ damage induced by cold preservation in *ex vivo* mouse liver and kidney models. The organ protective effects of PEP were greater than those of glucose or NAC, which are components of organ preservation solutions. Organ protection during cold preservation by PEP may be independent of antioxidative effects. These results suggest that PEP is a bifunctional carbohydrate metabolite with organ protective and antioxidant activity and has the potential for use as an agent for organ preservation in a clinical transplantation setting.
